# Preclinical Evaluation of Chicken Egg Yolk Antibody (IgY) Anti-RBD Spike SARS-CoV-2—A Candidate for Passive Immunization against COVID-19

**DOI:** 10.3390/vaccines10010128

**Published:** 2022-01-17

**Authors:** Hendris Wongso, Isa Mahendra, Wyanda Arnafia, Idar Idar, Muhammad Yusuf, Arifudin Achmad, Holis A. Holik, Ahmad Kurniawan, Iim Halimah, Maula E. Sriyani, Teguh H. A. Wibawa, Muhamad B. Febrian, Yanuar Setiadi, Eva M. Widyasari, Isti Daruwati, Crhisterra E. Kusumaningrum, Toto Subroto

**Affiliations:** 1 Research and Technology Center for Applied Nuclear, National Research and Innovation Agency, Jl. Tamansari No. 71, Lb. Siliwangi, Coblong, Bandung 40132, Indonesia; hendriswongso@batan.go.id (H.W.); isamahendra@batan.go.id (I.M.); ahmad-kurniawan@batan.go.id (A.K.); iimhalimah@batan.go.id (I.H.); maula@batan.go.id (M.E.S.); t_hafiz@batan.go.id (T.H.A.W.); mbasitf@batan.go.id (M.B.F.); yanuar-setiadi@batan.go.id (Y.S.); evamaria@batan.go.id (E.M.W.); isti@batan.go.id (I.D.); ellen@batan.go.id (C.E.K.); 2 Veterinary Pharmaceutical Industry, PT. Tekad Mandiri Citra, Jl. Mekar Raya Kav. 9, Bandung 40614, Indonesia; wyandaa@gmail.com; 3 Faculty of Pharmacy, Universitas Bhakti Kencana, Jl. Soekarno-Hatta No. 754, Bandung 40614, Indonesia; idar@bku.ac.id; 4 Department of Chemistry, Faculty of Mathematics and Natural Sciences, Universitas Padjadjaran, Jl. Raya Bandung Sumedang Km 21, Sumedang 45363, Indonesia; m.yusuf@unpad.ac.id; 5 Research Center for Molecular Biotechnology and Bioinformatics, Universitas Padjadjaran, Jalan Singaperbangsa No. 2, Bandung 40132, Indonesia; 6 Department of Nuclear Medicine and Molecular Theranostics, Faculty of Medicine, Hasan Sadikin General Hospital, Universitas Padjadjaran, Jl. Pasteur No. 38, Bandung 40161, Indonesia; a.achmad@unpad.ac.id; 7 Department of Pharmaceutical Analysis and Medicinal Chemistry, Faculty of Pharmacy, Universitas Padjadjaran, Jl. Bandung-Sumedang Km 21, Sumedang 45363, Indonesia; holis@unpad.ac.id

**Keywords:** IgY anti-RBD spike SARS-CoV-2, passive immunization, COVID-19, preclinical evaluation, radiolabeled IgY

## Abstract

The coronavirus disease 2019 (COVID-19) has become a substantial threat to the international health sector and the global economy. As of 26 December 2021, the number of mortalities resulting from COVID-19 exceeded 5.3 million worldwide. The absence of an effective non-vaccine treatment has prompted the quest for prophylactic agents that can be used to combat COVID-19. This study presents the feasibility of chicken egg yolk antibody (IgY) anti-receptor-binding domain (RBD) spike SARS-CoV-2 as a strong candidate to neutralize the virus for application in passive immunization. For the purpose of preclinical studies, we radiolabeled IgY anti-RBD spike SARS-CoV-2 with radionuclide iodine-131. This allowed us to evaluate several biological characteristics of IgY in vitro, in vivo, and ex vivo. The preclinical data suggest that IgY anti-RBD spike SARS-CoV-2 could specifically bind to the SARS-CoV-2 antigens; however, little uptake was observed in normal cells (MRC-5) (<2%). Furthermore, the ex vivo biodistribution study revealed that IgY predominantly accumulated in the trachea of normal mice compared to other organs. We also found that IgY possessed a good safety profile when used as an intranasal agent. Taken together, we propose that IgY anti-RBD spike SARS-CoV-2 has the potential for application in passive immunization against COVID-19.

## 1. Introduction

The ongoing coronavirus disease 2019 (COVID-19) is an extremely contagious and infectious viral illness induced by severe acute respiratory syndrome coronavirus 2 (SARS-CoV-2) [1]. The first cases of COVID-19 were announced on 31 December 2019 in Wuhan, China. Since then, COVID-19 has promptly developed into a global pandemic and become a substantial threat to international public health and the international economy [2,3]. As of 26 December 2021, the number of reported deaths due to COVID-19 is more than 5.3 million globally [4]. The main clinical signs of COVID-19 infection are fever, cough, pharyngitis, sore throat, dyspnea, fatigue, congestion and runny nose, asthenia and myalgia, nausea and vomiting, and diarrhea [5,6]. At present, a number of prophylactic and therapeutic treatments are being developed and repurposed for COVID-19, including passive immunization, viral drugs, and vaccines [7,8,9].

The COVID-19 pandemic remains a significant problem for the global society [10], and a new type of coronavirus variant, Omicron, has recently raised further concerns [11]. Most COVID-19 patients present mild and moderate symptoms (~80%) and recover with supportive care. Nevertheless, COVID-19 can cause severe and critical illness for some patients (~19%), such as acute respiratory distress syndrome (ARDS), metabolic acidosis, and a fatal drop in blood pressure [12,13,14,15]. In severe cases, the mortality rate can be up to 50% in adults [16]. According to the official data released by the World Health Organization (WHO), the global crude mortality rate is approximately 3.9% [17]. To date, numerous COVID-19 vaccines (e.g., Moderna, Johnson & Johnson/Janssen, Sinovac, Pfizer-BioNTech, and AstraZeneca) are available in many countries, and these can significantly reduce the incidence and/or severity of symptoms and the mortality rate; however, there are still concerns regarding possible reduced vaccine efficacy, especially in the context of rapid virus mutation [18,19]. In addition, the lack of potent non-vaccine treatment underlines concerns about possible future outbreaks of emerging virus-related ailments. Hence, effective prophylactic and therapeutic agents, particularly ones that could be stockpiled for future outbreaks, are urgently needed.

The necessity to combat SARS-CoV-2 has prompted investigations seeking drugs that neutralize the virus for application in passive immunization. The use of neutralizing antibodies can be effective in decreasing the severity of COVID-19 infection and provide a prospective alternative to the current approved treatments [20]. Previously, passive immunization employing pathogen-specific antibodies has shown promising results against various pathogens in both human and animal studies. Immunoglobulin Y (IgY), the main antibody from chicken egg yolk, may provide new approaches for non-vaccine countermeasures [21]. The efficacy and safety of IgY have been demonstrated in several reports. For example, prophylactic intra-oral IgY treatments have shown positive results in reducing chronic *Pseudomonas aeruginosa* manifestation in cystic fibrosis subjects [22,23]. Furthermore, IgY was found to be effective in blocking the internalization of *Staphylococcus aureus* into mammary epithelial cells, resulting in the deactivation of bacterial toxins [24]. In addition, chicken IgY possesses some advantages, such as the simple method for noninvasive local delivery by oral or intranasal administration, high specificity and avidity, limited adverse immune responses, capability to recognize more epitopes on a targeted antigen, the relatively quick production process, and high yield (40–80 mg antibody per egg) [25,26].

The preliminary investigation of the potential use of IgY for COVID-19 has been reported by few authors, mainly focused on the in vitro studies (e.g., neutralization assay and antiviral activities) [21,27,28]. These studies undoubtedly have contributed to current knowledge, and may provide the practical basis for the development of chicken egg yolk IgY for passive immunization against COVID-19. Apart from these, comprehensive in vitro, in vivo, and ex vivo studies of IgY as a prophylactic agent for COVID-19 have not been described previously. Here, we report the preclinical data of IgY anti-receptor-binding domain (RBD) spike SARS-CoV-2 as a promising candidate for passive immunization against COVID-19. In this study, we also employed a radiotracer technique to evaluate several biological properties of IgY in a fast and efficient manner. Further studies including in vivo evaluation of IgY anti S-RBD spike SARS-CoV-2 in animals infected by SARS-CoV-2 and clinical trials will be initiated in the near future.

## 2. Materials and Methods

### 2.1. Materials

High-purity chemicals were purchased from Merck, Singapore (2 Science Park Drive, Singapore) and used without further purification. [^131^I]Na was obtained from G.A. Siwabessy Multipurpose Reactor (PUSPIPTEK, Serpong, Indonesia) and Hasan Sadikin General Hospital (Bandung, Indonesia). The human lung cell line (MRC-5) was purchased from Elabscience (Wuhan, China). The cell line was cultured in minimum essential media (MEM) (Gibco, Cat.no. 11095080) with fetal bovine serum (Sigma Aldrich, St. Louis, MO, USA; Cat.no. F2442) and penicillin-streptomycin solution (Sigma Aldrich, St. Louis, MO, USA; Cat.no. C61664) added. A His-tagged recombinant protein of SARS-CoV-2 S-RBD was purchased from Thermo Fisher Scientific (Invitrogen, Cat.no. RP-87678; Waltham, MA, USA). The radioactivity was measured using a dose calibrator (Biodex; Shirley, NY, USA) and automatic gamma counter with a well-type NaI(Tl) detector (2470 Wizard2™, Perkin Elmer; Waltham, MA, USA). Radio-thin layer chromatography (radio-TLC) analysis was performed on a Bioscan AR-2000 (Washington, DC, USA). Paper electrophoresis was performed on a cellulose acetate membrane, 60 mm × 220 mm (Major Science Mini-300; Saratoga, CA, USA). High-performance liquid chromatography (HPLC) analysis was conducted on a UV and a GABI raytest radioactivity detector (Agilent Technologies, 1200 Infinity Series; Santa Clara, CA, USA) using an analytical size exclusion chromatography (SEC) column (Agilent SEC 3 300A 70.8 mm × 300 mm; St. Clara, CA, USA). The shaker incubator model was Thermo TS-100C. Purification of the radiolabeled IgY was carried out using Sephadex G-25 Fine (Merck, Singapore). Human serum was obtained from the Indonesian Red Cross Society. Animal studies were performed on normal mice (6-week-old BALB/c male and female mice (weight ~30 g)) purchased from Bogor Life Science and Technology Ltd., Bogor, Indonesia. Mice were acclimated for 7 days before being given the treatment. The housing system was set at 26 °C and 30% to 60% relative humidity with a photoperiod of 12:12.

### 2.2. Immunization of Chicken and Egg Yolk Collection

Immunization of chickens was carried out by injecting 0.5 mL (per chicken) of commercially RBD of SARS-CoV-2 antigen (GenScript-China; Nanjing, Jiangsu, China) and adjuvant (complete Freund’s adjuvant) via intramuscular in the musculus pectoralis. Furthermore, boosting was given every 14 days (with the same dose of the vaccine and incomplete Freund’s adjuvant). Chicken serum and eggs were collected before the first vaccination and seven days after each boosting. The yolk of the egg was separated from egg white using an egg separator and filter paper. During the immunization period, chickens were maintained in controlled environments: the cage size was 35 cm × 35 cm × 37.5 cm per chicken. The house temperature ranged from 23–25 °C with humidity around 55–60%. The lighting system used lights with a light-dark cycle, light for 14 h and dark for 10 h. Air circulation was equipped with an inlet to suck air and exhaust to remove air. The cooling system was supported by an air conditioner. Chickens were fed and drank ad-libitum [29,30].

### 2.3. Extraction of IgY Anti-RBD Spike SARS-CoV-2

IgY anti-RBD spike SARS-CoV-2 was extracted from egg yolks using the PEG precipitation method, as described previously with minor modifications [31]. To a certain volume of egg yolk was added 2× volume of phosphate buffer saline (PBS) (pH = 7.5), followed by fine solid of polyethylene glycol-6000 (PEG-6000) to give a final concentration of 3.5% (*w*/*v*). After being stirred and incubated at room temperature (rt) for 20 min, the solution was centrifuged (13,000× *g*; 20 min; 4 °C). The resulting supernatant was collected and filtered through a coarse filter paper. Subsequently, it was added by PEG-6000 to a concentration of 8.5% (*w*/*v*), stirred, and centrifuged as described above. The precipitate was resuspended, and another precipitation step was performed with 12% of PEG-6000 (*w*/*v*).

### 2.4. Purification of IgY Anti-RBD Spike SARS-CoV-2

Purification of IgY was accomplished using the AKTA Start chromatography system with HiTrap IgY Purification HP column. The precipitate resulting from PEG precipitation was dialyzed against equilibration buffer pH 7.5 (20 mM sodium phosphate, 0.5 M potassium sulphate). A dialyzed sample was subjected to the AKTA Start chromatography system, which was equilibrated by four resin-bed volumes of equilibration buffer. The column was washed with 5–10 resin-bed volumes of equilibration buffer. Then, the bound IgY was eluted with 12 resin-bed volumes of elution buffer (20 mM sodium phosphate, pH 7.5), and the eluent was collected in 3 mL fractions. The column was regenerated with pH 7.5 (20 mM sodium phosphate, 30% propan-1-ol) regeneration buffer. All running process in the AKTA chromatography system has a flow rate of 1.0 mL/min. Purified IgY anti-RBD spike SARS-CoV-2 was characterized by sodium dodecyl sulphate–polyacrylamide gel electrophoresis (SDS-PAGE) [32,33], and protein concentration was measured by Lowry’s method [34].

### 2.5. Labeling Chemistry

The radiolabeled IgY anti-RBD spike SARS-CoV-2 ([^131^I]-IgY) was prepared according to the general antibody radioiodination procedure published by Gupta and colleagues [35], with modifications. Briefly, iodogen (5.0 mg) was dissolved in a polypropylene microcentrifuge tube containing chloroform (0.5 mL), and the solution was slowly evaporated under a stream of nitrogen to prepare an iodogen-coated tube. IgY anti-RBD spike SARS-CoV-2 (0.5 mg in PBS 0.5 M) was added to the solution of PBS 0.5 M (250 µL) in an iodogen-coated tube, followed by aqueous [^131^I]Na (9.25–11.1 MBq). The reaction mixture was incubated in a microtube shaker (150 rpm) at rt for 2 min. Then, 50 µL of sodium metabisulfite in PBS 0.5 M (1.0 mg/mL) was added to the tube to give [^131^I]-IgY. The radiolabeled product was subjected to a buffer-equilibrated (PBS 0.5 M) Sephadex G-25 column to remove the free iodine from the radioiodinated IgY. PBS 0.5 M was added to load the column reservoir, and 30 × 400 µL fractions were collected into 1.0 mL polypropylene tubes. Subsequently, the radioactivity in each fraction was measured by a dose calibrator. The first peak represents [^131^I]-IgY, while the following flat peak reflects free iodine. The radiochemical purity of [^131^I]-IgY was assessed by radio-TLC, paper electrophoresis, and HPLC. Radio-TLC was carried out on silica gel iTLC-SG 10 cm and eluted with methanol/water (75:25%, *v*/*v*). Paper electrophoresis was carried out on a cellulose acetate membrane filter, 60 mm × 220 mm, for 1 h at a voltage of 300 V using buffer phosphate 0.1 M (pH 7.4) as an electrolyte source. For TLC and electrophoresis analyses, the percentage of labeling was evaluated using a radio-TLC scanner. Analytical HPLC was performed using an SEC column as stationary phase. The HPLC conditions were as follows: A = 1% TFA/H_2_O; B = 1% TFA/acetonitrile; UV-detection at 280 nm; isocratic elution for 30 min (A:B, 50/50%); and the flow rate was 1.0 mL/min.

### 2.6. Stability Study

The stability of the purified [^131^I]-IgY anti-RBD spike SARS-CoV-2 was evaluated by measuring the radiochemical purity (RCP). The RCP was determined by radio-TLC after remaining in PBS (0.5 M) and human serum at rt (~26 °C) for 0, 30, 60, 150, and 180 min, and 24, 48, and 72 h, respectively, with triplicates performed for each condition.

### 2.7. Lipophilicity Measurement

To measure the lipophilicity (log *P*) of [^131^I]-IgY anti-RBD spike SARS-CoV-2, 100 µL of the radiolabeled antibody was mixed with 900 µL of NaCl (0.1% in H_2_O, *w*/*v*) and 1.0 mL of 1-octanol in a centrifuge tube (*n* = 3). The solution was agitated on a vortex mixer at rt for 30 s, and centrifuged at 3000 rpm for 15 min. The water and 1-octanol layers were separated, and the radioactivity of each fraction was determined with a dose calibrator. The log *P* was calculated as follows: *P* = (count per min in 1-octanol/count per min in NaCl).

### 2.8. Cellular Uptake Study

The MRC-5 cell line (1.0 × 10^5^ cells/well) was seeded into 24-well culture plates in complete medium (MEM containing fetal bovine serum and penicillin–streptomycin) overnight. The medium was separated, and the cells were rinsed with a solution of Hank’s balanced salt solution (HBSS). Subsequently, [^131^I]-IgY anti-RBD spike SARS-CoV-2 (0.5–0.7 MBq; 27–31 µg IgY in 10 µL solution) was added into each well containing HBSS and incubated at 37 °C for 10, 30, and 60 min. Then, the cells were washed with HBSS and lysed using a sodium hydroxide solution (0.2 M). The cell lysates were analyzed using an automatic gamma counter with a well-type NaI(Tl) detector to determine the radioactivity. Results of cellular uptake was expressed as a percentage of cellular uptake, as determined by the formula below:

$$\%\;\mathrm{uptake}=\frac{\mathrm{Count}\;\mathrm{value}\;\mathrm{of}\;\mathrm{cell}\;\mathrm{lysates}}{\mathrm{Count}\;\mathrm{value}\;\mathrm{of}\;a\;3.7\;\mathrm{kBq}\;\mathrm{of}\;\mathrm{radiolabeled}\;\mathrm{antibody}\;}\times 100\%$$

### 2.9. Magnetic Bead Assay

The magnetic bead assay was carried out as previously outlined [36,37] to determine IgY immunoreactivity. The study design consisted of three arm groups (*n* = 5)—a control with no recombinant protein S-RBD as the antigen, a positive control with recombinant protein S-RBD, and a blocking control using excess IgY anti-RBD spike SARS-CoV-2. The nonspecific binding of the [^131^I]-IgY to beads lacking the S-RBD was determined in the first arm group as a control; the second arm determined [^131^I]-IgY binding to the S-RBD His-tagged recombinant protein, and the third arm demonstrated the specificity of radioligand binding to a similar recombinant protein in the presence of an excess of unlabeled IgY anti S-RBD.

Briefly, a 20 µL volume of a Ni-NTA magnetic bead slurry (HisPur™ Ni-NTA magnetic beads, Thermo Scientific; Waltham, MA, USA) was placed into a 1.5 mL LoBind microcentrifuge tube. The beads were then rinsed with a solution of PBS containing 1% bovine serum albumin (BSA), followed by brief homogenization in a vortex mixer for 5 s before being placed on a DynaMag™-2 magnetic rack (Thermo Scientific; Waltham, MA, USA) for 45 s. The magnetic beads were isolated after discarding the supernatant. The washed beads were then resuspended in 390 µL of PBS-BSA. The beads in the second and third arms were incubated with 1 µg of SARS-CoV-2 S-RBD His-tagged recombinant protein (Invitrogen; Waltham, MA, USA) on a thermomixer at 300 rpm for 15 min. Thereafter, the beads were rinsed with PBS-T before incubation with the radiolabeled [^131^I]-IgY anti-RBD spike SARS-CoV-2 in PBS-BSA for 30 min on rotating mixer. Excess unlabeled IgY anti S-RBD was added in the third arm a few seconds prior to adding the radioligand. Subsequently, the supernatant containing unbound [^131^I]-IgY was separated using a magnetic rack. The beads were then washed twice using PBS-BSA to remove nonspecifically bound radioligands. Finally, the radioactivity in the beads was measured using a well-type automatic gamma counter. The relative binding fraction (RBF) was expressed as the percentage of radioactivity attached to the magnetic beads to the total radioactivity.

### 2.10. Biodistribution and Blood Clearance Studies

A solution of [^131^I]-IgY anti-RBD spike SARS-CoV-2 (0.5–0.7 MBq/10 µL) was administered via the intranasal route. The mice were euthanized at 10 min, 30 min, 1 h, 24 h, and 72 h after administration. The radioactivity of 16 organs from each mice was analyzed using an automatic gamma counter with a well-type NaI(Tl) detector. The [^131^I]-IgY biodistribution was expressed as the percentage of injected radioactivity dose (% ID) or % ID per organ weight (% ID/g).

Blood clearance studies were applied to the [^131^I]-IgY anti-RBD spike SARS-CoV-2 and iodine-131 (*n* = 5). The mice were intranasally administered 0.5–0.7 MBq/10 µL of [^131^I]-IgY (27–31 µg) or iodine-131. At specified times of 5 min, 30 min, 1 h, 2 h, 24 h, 25 h, 48 h, and 49 h post-administration, blood samples were drawn from the tail of each mouse. The blood samples were then weighted and counted using an automatic gamma counter with a well-type NaI(Tl) detector. The measurement result was processed as % ID per blood weight (% ID/g). Half-life was calculated from the exponential decay by nonlinear regression using GraphPad Prism 8.4.3 software.

### 2.11. Acute Toxicity Study

An acute toxicity study of IgY anti-RBD spike SARS-CoV-2 was performed according to WHO and OECD guideline 423. Female and male mice were fasted for 3 h before intranasal administering of IgY anti S-RBD. In this toxicity study, 3 groups of IgY doses were utilized (0.5, 50, and 500 mg/kg body weight (BW)). Animals were individually observed at 30 min after administration prior to regular observation throughout the first 24 h for a total of 14 days. The clinical symptoms, mean weight gain, gross pathology, histopathology, and LD_50_ were evaluated.

### 2.12. Statistical Analyses

Data were provided as mean ± standard deviation (SD), unless otherwise noted. The statistical analyses were carried out utilizing the GraphPad Prism 8.4.3 software (GraphPad Software, La Jolla, CA, USA). The significance within the multiple groups was determined by one-way analysis of variance (ANOVA), followed by Tukey’s test. The data were considered statistically different if the *p* value ≤ 0.05.

## 3. Results

### 3.1. Extraction and Purification of IgY Anti-RBD Spike SARS-CoV-2

**Figure 1:**
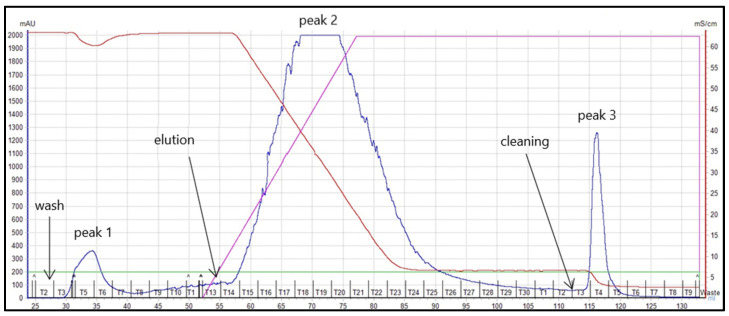
Separation of dialyzed IgY anti-RBD spike SARS-CoV-2 using chromatography system, AKTA START™ on a HiTrap IgY Purification HP column. Flow-through was pool fractions 3–6 (peak 1); eluate was pool fractions 15–24 (peak 2); cleaning or column regeneration (peak 3). Absorbance intensity of protein (mAU), solution conductivity (mS/cm), and solution gradient concentration are shown in blue, red, and purple lines, respectively.

IgY anti-RBD spike SARS-CoV-2 was successfully isolated from egg yolks using the PEG precipitation method. PEG-6000 at concentration of 3.5% (*w*/*v*) was used to precipitate the lipids from the yolk, while PEG-6000 12% (*w*/*v*) precipitated the IgY anti-RBD spike SARS-CoV-2. Purification of dialyzed IgY anti-RBD spike SARS-CoV-2 using chromatography system, AKTA START™, with a HiTrap IgY Purification HP column, was resulted in three protein peaks (Figure 1).

**Figure 2:**
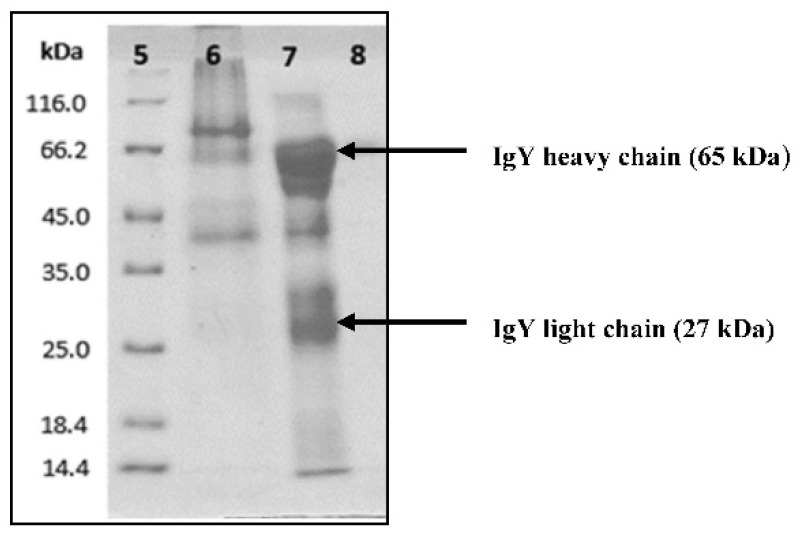
Identification and SDS-PAGE patterns of IgY anti-RBD spike SARS-CoV-2. Protein marker (lane 5), peak 1 (lane 6), peak 2 (lane 7), and peak 3 (lane 8).

The purity and molecular identity of each peak was characterized using SDS-PAGE. IgY anti-RBD spike SARS-CoV-2 has a molecular weight approximately 180 kDa. Under the reducing condition, the disulfide bridge of IgY anti-RBD spike SARS-CoV-2 would cleave into two fragments, consisting of two heavy chains (65 kDa) and two light chains (27 kDa). Flow-through (peak 1), generated when applying the equilibrium buffer, was confirmed as impurities having neither 65 nor 27 kDa band. Peak 2, yielding 65 and 27 kDa proteins bands, was confirmed as IgY anti-RBD spike SARS-CoV-2. Peak 3, produced from the column cleaning step, did not have 65 and 27 kDa protein bands (Figure 2). Therefore, peak 2 was considered as pure IgY anti-RBD spike SARS-CoV-2, and used for further studies. The protein concentration of purified IgY anti-RBD spike SARS-CoV-2 was ~20 mg per egg, as determined by Lowry’s method.

### 3.2. Radiolabeling

**Figure 3:**
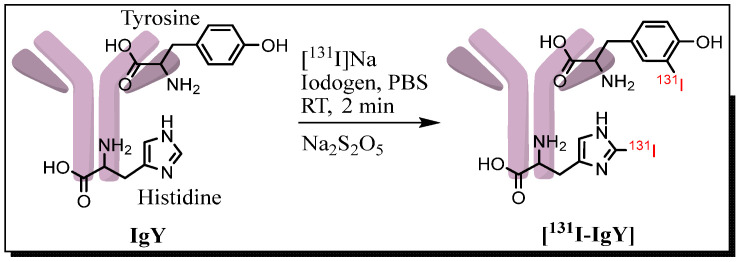
Schematic of direct radioiodination of IgY with iodine-131.

The radioiodination of IgY anti-RBD spike SARS-CoV-2 was accomplished through a rapid and efficient reaction, providing a radiochemical yield (RCY) of 96.6 ± 0.6 (*n* = 3) after 2 min of incubation. Iodogen was used as an oxidizing agent to facilitate direct conjugation of positive radioiodine species (I^+^) into tyrosine and/or histidine residues in the IgY anti-RBD spike SARS-CoV-2 structure. In large biomolecules, such as proteins and antibodies, the primary site responsible for iodination is tyrosine residues; however, in more basic reaction conditions (pH exceeds 8.5), the secondary site on the histidine residues (imidazole ring) is favored (Figure 3) [38,39].

**Figure 4:**
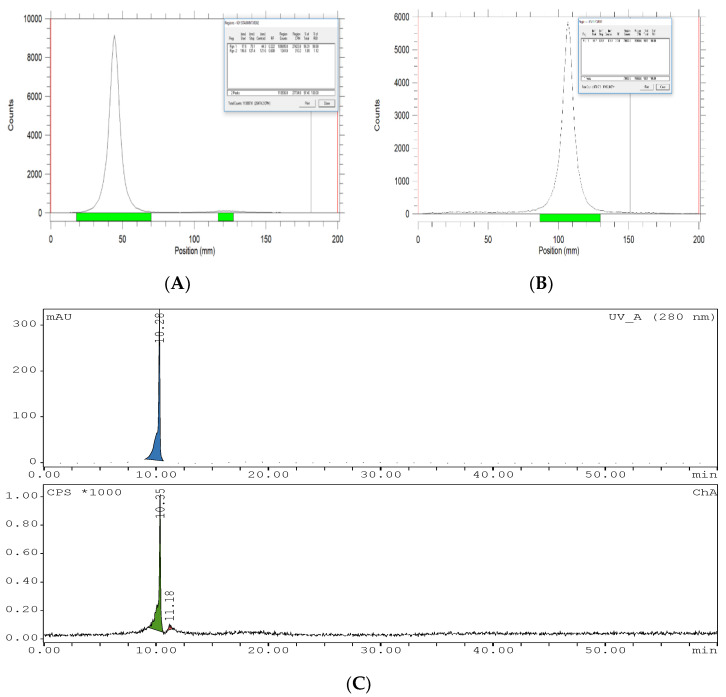
Representative radio-TLC chromatogram of [^131^I]-IgY anti-RBD spike SARS-CoV-2 (**A**), electropherogram of [^131^I]IgY anti-RBD spike SARS-CoV-2 (**B**), and HPLC profiles of [^131^I]-IgY anti-RBD spike SARS-CoV-2: UV detector (above) and radioactive detector (below) (**C**).

Purification using SEC gave a radiochemical purity of 99.8 ± 0.3 (*n* = 3) (TLC analysis) (Figure 4A). The TLC results indicated that free iodine moved with the solvent front, while the radiolabeled IgY remained at the origin. To validate the radio-TLC, paper electrophoresis and radio-HPLC of [^131^I]-IgY were carried out, and it was confirmed that no fragmentation or decomposition occurred after purification. Paper electrophoresis showed that [^131^I]-IgY remained at the spotting point (centroid) (Figure 4B). Analysis of the HPLC indicated that the retention time (RT) of [^131^I]-IgY was 10.35 min (Figure 4C), lower than the standard IgY (RT 12.28 min) (Supplementary Materials, Figure S1). Due to the introduction of iodine atoms, the size and molecular weight of the radiolabeled IgY was increased, and therefore passed through the column faster and eluted first. Free iodine was observed at RT 11.18 min.

### 3.3. Physicochemical Properties Evaluation

#### 3.3.1. Stability

**Figure 5:**
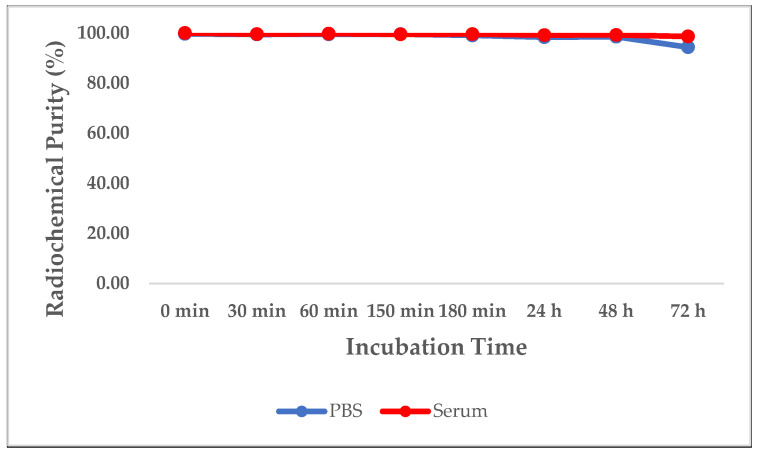
Radiolabeling stability of [^131^I]-IgY anti-RBD spike SARS-CoV-2 in PBS and human serum over 72 h.

To assess the stability of [^131^I]-IgY anti-RBD spike SARS-CoV-2, the compound was incubated with a solution of PBS (0.5 M) and human serum at rt. According to the radio-TLC analysis, the radiolabeled IgY remained stable for 72 h after treatment with PBS (0.5 M) (RCP > 94%) and human serum (RCP > 98%). Almost no fragmentation of the [^131^I]-IgY was found, indicating its good in vitro stability (Figure 5).

#### 3.3.2. Partition Coefficient

The octanol/water partition coefficient (log *P*) of [^131^I]-IgY anti-RBD spike SARS-CoV-2 was determined and found to be −2.66 ± 0.28 (Supplementary Material Table S1). This relatively low log *P* value indicates the hydrophilic nature of IgY.

### 3.4. In Vitro Studies

#### 3.4.1. Cellular Uptake

**Figure 6:**
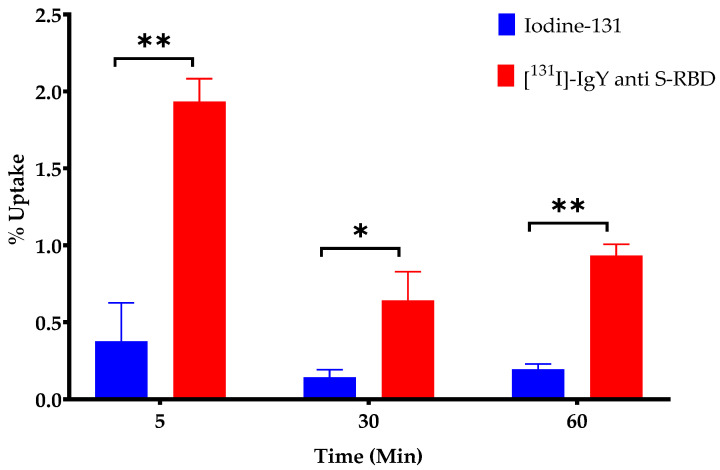
Cellular uptake of [^131^I]-IgY anti-RBD spike SARS-CoV-2 and iodine-131 (control) in MRC-5 cell line at 5, 30, and 60 min (*n* = 5). * *p* < 0.05, ** *p* < 0.01 compared with control group (iodine-131).

Prior to conducting the in vivo studies, we performed a cellular uptake study to determine the potential of IgY anti-RBD spike SARS-CoV-2 for COVID-19 treatment. The cellular uptake of iodine-131 (control) and [^131^I]-IgY anti-RBD spike SARS-CoV-2 in an MRC-5 cell line is shown in Figure 6. Although the cellular uptake of [^131^I]-IgY anti-RBD spike SARS-CoV-2 was higher than that of iodine-131 (*p <* 0.05), the accumulation of radiolabeled IgY in the MRC-5 cell line was considerably low (<2.0%), and gradually decreased until 60 min of incubation. These results may provide an indication that the [^131^I]-IgY anti-RBD spike SARS-CoV-2 is not specifically accumulated into normal lung cells, although further studies are needed to confirm this.

#### 3.4.2. Magnetic Bead Assay

**Figure 7:**
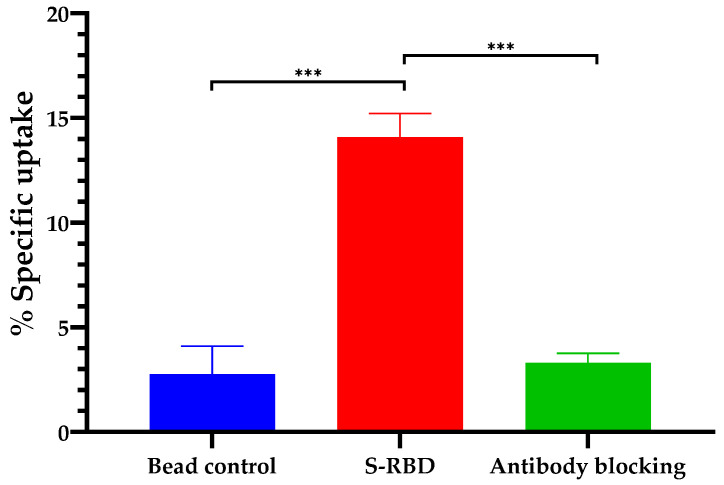
The specific uptake of [^131^I]-IgY anti-RBD spike SARS-CoV-2. Significant differences among the three arms are indicated as *** *p* < 0.001.

The specificity of IgY anti-RBD spike SARS-CoV-2 for the virus was investigated using a magnetic bead assay. The S-RBD of SARS-CoV-2 with a histidine tag was used as the antigen in the assay. Our results show that the uptake of [^131^I]-IgY by magnetic beads was significantly higher than the uptake in the group preincubated with IgY (Figure 7). These results confirm that the labeling process of the IgY with iodine-131 might not alter the active site to the S-RBD protein. The assay results also suggest that both IgY and [^131^I]-IgY are specific for S-RBD SARS-CoV-2.

#### 3.4.3. Ex Vivo Biodistribution

**Figure 8:**
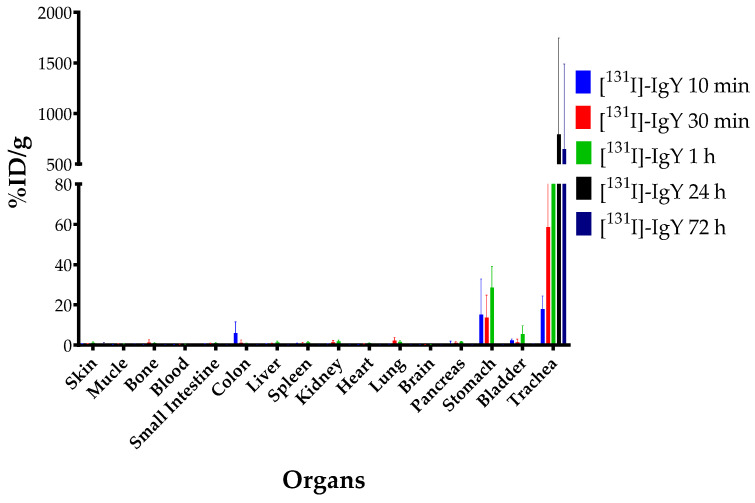
Organ accumulation of [^131^I]-IgY anti-RBD spike SARS-CoV-2 in normal mice at five time points post intranasal administration, presented as percentage of injection dose per gram of organ (% ID/g).

For the ex vivo biodistribution study, IgY anti-RBD spike SARS-CoV-2 was radiolabeled with iodine-131. This allowed us to evaluate the distribution of IgY in normal mice by measuring the radioactivity accumulated in each organ. Figure 8 describes the biodistribution of [^131^I]-IgY in the major organs, presented as the percentage of injected dose (% ID) divided by a snippet of organ weight (gram). The highest accumulation of [^131^I]-IgY was found significantly in the trachea 24 h post intranasal administration (794.70%ID/g). A small portion of [^131^I]-IgY was found in the stomach, with the highest accumulation at 1 h post intranasal administration (28.57%ID/g). All organs showed a similar pattern of accumulation increment from 10 min to 1 h before significantly decreasing at 24 h. The accumulation of [^131^I]-IgY in the trachea was found to gradually increase over time, with the highest percentage observed in 24 h after intranasal administration and slightly decreased at 72 h.

**Figure 9:**
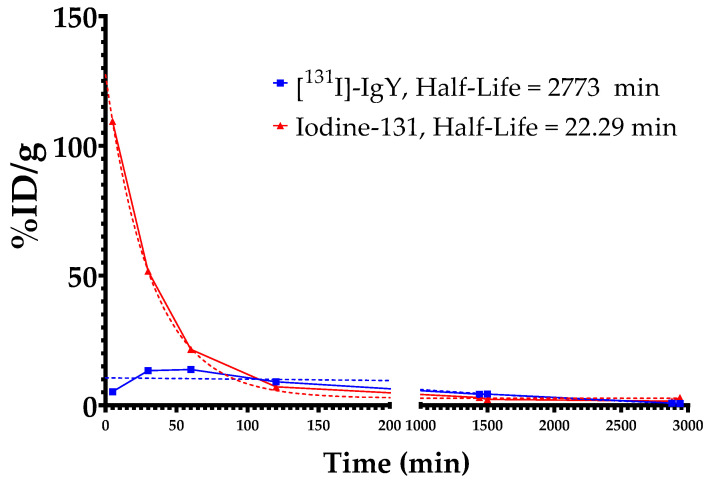
Blood clearance profile of [^131^I]-IgY anti-RBD spike SARS-CoV-2 expressed as exponential decay.

The rate of the clearance of [^131^I]-IgY anti-RBD spike SARS-CoV-2 in the blood was predicted by measuring its half-life at 5, 30 min, 1, 2, 24, 25, 48, and 49 h. The results were compared with the half-life of the iodine-131 as control. This study demonstrated that the half-life of [^131^I]-IgY was found to be 2773 min, which is three times higher than that of the control (Figure 9).

#### 3.4.4. Acute Toxicity Study

**Figure 10:**
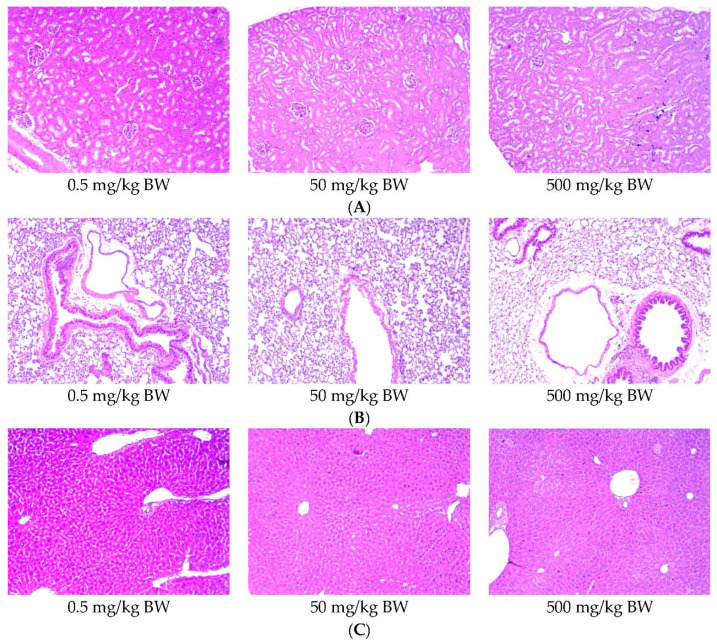
Histology of kidney (**A**), lung (**B**), and liver (**C**) of mice after intranasal administration of IgY anti SARS-CoV-2 at different doses (magnification: 100x).

The acute toxicity studies of IgY anti-RBD spike SARS-CoV-2 showed that the animals were free of intoxicating signs during 14 days after intranasal administration. None of mice died until they were administered a dose of 500 mg/kg BW, as described in Table 1. No mice showed clinical symptoms, such as convulsion, diarrhea, dyspnea, and Straub signs (Table 2). Furthermore, we did not find any changes in the gross pathology in a macroscopical assessment of the organs, especially in the kidney, lung, and liver (Figure 10).

## 4. Discussion

This study presents a comprehensive preclinical evaluation of IgY anti-RBD spike SARS-CoV-2 for its potential application in COVID-19 passive immunization. The IgY antibody was produced from the egg yolk of chickens immunized using the virus RBD as antigen in high purity (>90%) and yields (~20 mg per egg), with minimal environmental harm and infrastructure investment. Analysis of the SDS-PAGE showed two molecular patterns at 65 kDa and 27 kDa, corresponding to the IgY heavy and light chains, respectively [26]. We futher radiolabeled the IgY with iodine-131 to allow us to investigate several in vitro (e.g., immunoreactivity, cellular uptake) and in vivo (e.g., biodistribution, pharmacokinetic) parameters. The radiolabeling technique was found to be effective in drug development application because of its simple detection and analysis [40].

Radiolabeling IgY with iodine-131 was accomplished by direct radioiodination using the iodogen method [35] to prepare [^131^I]-IgY anti-RBD spike SARS-CoV-2. Iodine-131 is a very good choice due to its ideal nuclide properties (beta and gamma emitters), physical half-life (8.04 days), availability, and affordability [41]. The [^131^I]-IgY was then purified using a size exclusion column (Sephadex G-25) and characterized. The purified [^131^I]-IgY had an RCP of >99.0% and was stable up to 72 h post-radioiodination without loss of integrity, both in PBS and human serum. The lipophilicity of the IgY was predicted using the partition coefficient (log *P*) in octanol/water and was found to be −2.66 ± 0.28. This low log *P* value indicated the hydrophilic nature of the IgY, which is very common for antibodies [42], and the presence of the iodine atoms did not significantly alter the log *P*.

Recent studies on the development of immunoprophylaxis for COVID-19 have aimed to establish RBD-specific blocking of SARS-CoV-2 by preventing virus infection via the ACE2 receptor [43]. An in vitro study revealed that RBD-specific IgY has the ability to inhibit the initial binding of the viral spike glycoprotein to human host receptors (ACE2) in addition to viral replication [21]. Similarly, the study also showed that IgY exhibited neutralization activity against pseudotyped and live SARS-CoV-2 [27]. Interestingly, the essential parameters, including cellular uptake, biodistribution, in vivo clearance, and toxicity have not been described previously, especially in the context of IgY anti-RBD spike SARS-CoV-2 for COVID-19. Thus, our study may provide new and valuable information in COVID-19-related research.

The effectiveness of the administration route for IgY has been reported in normal mice using in vivo fluorescent imaging techniques. It was found that IgY antibodies administrated via an oral spray or nasal drip remained in the upper airways for up to 24 h [27]. Additionally, the nasal and olfactory mucosa are surrounded with ACE2 receptors, making the upper airways susceptible to COVID-19 infection [44]. Moreover, the strategy of intranasal administration of IgY for passive immunization was applied for *P. aeruginosa* infection in the trachea. The results demonstrated that specific anti-*P. aeruginosa* IgY transiently reduces bacteria aggregation in the airway of mechanically ventilated piglets [45]. Therefore, in our study, the administration of IgY anti-RBD spike SARS-CoV-2 was designed to occur via the intranasal route to permit favorable accumulation in the upper respiratory tracts.

To investigate the specificity of IgY S-RBD to the SARS-CoV-2 antigen target, an immunoreactivity study using a magnetic bead assay was carried out. The results demonstrated that both unlabeled and radiolabeled IgY specifically bound to the S-RBD protein. This also suggests that the labeling process of IgY with iodine-131 did not change the active site for binding to the S-RBD protein (Figure 7). The data were in line with previously reported results, where IgY from chickens immunized with inactivated SARS-CoV-2 attached to the RBD in S1 in a dose-dependent manner and thereby prevented adhesion between ACE2 and RBD [27].

From the ex vivo biodistribution study, it was revealed that IgY anti-RBD spike SARS-CoV-2 persisted in the trachea, and the accumulation gradually increased up to 24 h after intranasal administration. The radiolabeled IgY showed a half-life of 2773 min, which indicates its slow elimination from the body (Figure 9), associated with low uptake by the kidney. Thus, a high trachea uptake may lead to slow excretion of the IgY. High tracheal accumulation of IgY was a good indicator for the development of passive immunization against COVID-19. It has been suggested that the trachea is an important organ during COVID-19 manifestation. In a cohort study of COVID-19-induced ARDS syndrome, all patients (100%) tested positive for COVID-19 in a tracheal aspirated test, while oropharyngeal swab tests in the same samples only accounted for a 77% positivity rate [46]. Epithelial cells in the trachea are among the first cell types to encounter viral infection via airways. This event may lead to trachea inflammation. Hence, the size of the trachea during SARS-CoV-2 infection has been proposed as a prognosis factor for COVID-19 patients [47]. An in vivo assay of human hyperimmune immunoglobulin COVID-19 therapy (convalescence plasma therapy) showed that intravenous injection of human immunoglobulin in a hamster model of SARS-CoV-2 could reduce viral replication in the trachea and the lung [48].

On the other hand, IgY anti-RBD spike SARS-CoV-2 uptake in the lung of normal mice was quite low (Figure 8) because our IgY was designed to specifically attach to the S1 RBD protein of SARS-CoV-2 instead of ACE2 receptors. Accumulation in the lung was consistent with the results of in vitro studies using the MRC-5 lung cell line, which showed low accumulation (<2%) and gradually decreased over 1 h. IgY also accumulated in the stomach, and bladder in relatively high % ID/g values compared to other organs, particularly 1 h after intranasal administration. These accumulations may be related to IgY escape from the nasal cavity into the digestive tract. A similar uptake pattern for the stomach was found in a study of orally administered IgY as a passive vaccine for orogastrointestinal diseases [49]. Furthermore, IgY accumulation in the blood was relatively low and remained basically constant over 72 h. The low uptake in the blood could ensure that intranasal administration of IgY only targeted the respiratory system, allowing non-target uptake by other organs to be minimized.

We performed an acute toxicity assessment of the IgY anti-RBD spike SARS-CoV-2 in normal mice. In this experiment, the animals were divided into groups that received IgY at three different doses (0.5, 50, and 500 mg/kg BW). No deaths were observed in any of the animal groups. Moreover, intranasal administration of IgY did not cause abnormal clinical symptoms (Table 1 and Table 2) or gross pathology and histopathology changes in the vital organs, including the kidney, lung, and liver (Figure 10). These results confirmed the safety of IgY anti-RBD spike SARS-CoV-2 as a candidate for passive immunization against COVID-19.

Furthermore, reports have shown that IgY did not increase the level of cytokines, such as tumor necrosis factor α (TNF-α), granulocyte-macrophage colony-stimulating factor (GM-CSF), interleukin (IL)-1β, and IL-6 [50]. This is indicative of the potential use of IgY as a good candidate in therapy for COVID-19 patients with ARDS.

## 5. Conclusions

The results of this study indicate that chicken egg yolk antibody (IgY) anti-RBD spike SARS-CoV-2 could be labeled with iodine-131 with a high yield, purity, and stability and without the loss of product integrity. Purified [^131^I]-IgY with an RCP of >99% was further used for preclinical studies. The in vitro studies revealed that the radiolabeled and unlabeled IgY could interact with SARS-CoV-2 antigens; however, little interaction with normal lung cells (MRC-5) was observed. In addition, the biodistribution study demonstrated that IgY predominantly accumulated in the trachea compared to other organs. Moreover, the acute toxicity studies of IgY demonstrated that there were no signs of morbidity or death in mice after two weeks of treatment, and IgY can be safely administrated intranasally. Furthermore, IgY anti-RBD spike SARS-CoV-2 can be produced on a large scale with low costs and minimum infrastructure investment. Thus, the work presented here suggests the potential of IgY anti-RBD spike SARS-CoV-2 for further evaluation as a strong antibody candidate to be used in passive immunization via nasal administration to prevent the development of COVID-19. However, neutralization assays and in vivo studies using SARS-CoV-2-infected animal models need to be performed to confirm its efficacy.

## Figures and Tables

**Table 1:** Number of dead mice and LD_50_ for 14 days of observation in the acute toxicity study of IgY anti-RBD spike SARS-CoV-2.

Group/Dose (mg/kg BW)	n	Number of Dead (% Response)		LD_50_ Cut Off (mg/kg BW)
		Male	Female	
0.5	5	0	0	>500
50	5	0	0	
500	5	0	0	

**Table 2:** The clinical symptoms for 14 days of observation in the acute toxicity study of IgY anti-RBD spike SARS-CoV-2.

Group/Dose (mg/kg BW)	Clinical Symptoms of Toxicity					
	Convulsion	Diarrhea	Cornea Reflex	Dyspnea	Righting Reflex	Straub
0.5	-	-	+	-	+	-
50	-	-	+	-	+	-
500	-	-	+	-	+	-

## Data Availability

The data are contained within the article.

## Supplementary Materials

The following materials are available online at https://www.mdpi.com/article/10.3390/vaccines10010128/s1. Figure S1: HPLC chromatogram of the standard IgY (RT = 12.28 min); Figure S2: Representative TLC chromatograms for stability studies of [^131^I]-IgY anti-RBD spike SARS-CoV-2 in PBS over 72 h; Figure S3: Representative TLC chromatograms for stability studies of [^131^I]-IgY anti-RBD spike SARS-CoV-2 in human serum over 72 h; Figure S4: Identification and SDS-PAGE patterns of IgY anti-RBD spike SARS-CoV-2, and Table S1: Lipophilicity (Log *P*) of [^131^I]-IgY anti-RBD spike SARS-CoV-2.
