# Supplementary material for: Preclinical Evaluation of Chicken Egg Yolk Antibody (IgY) Anti-RBD Spike SARS-CoV-2—A Candidate for Passive Immunization against COVID-19
Source: Vaccines (Basel). 2022 Jan 17;10(1):128. doi: 10.3390/vaccines10010128 (PMC8778912; doi:10.3390/vaccines10010128)
Supplement: Supplementary file 1 [file vaccines-10-00128-s001.zip › vaccines-1501129-supplementary.pdf]

vaccines

Supplementary Materials: Preclinical Evaluation of Chicken Egg Yolk Antibody (IgY) Anti-RBD Spike SARS-CoV-2 – A Candidate for Passive Immunization against COVID-19

Hendris Wongso [1], Isa Mahendra [1], Wyanda Arnafia [2], Idar Idar [3], Muhammad Yusuf [4,5], Arifudin Achmad [6], Holis A. Holik [7], Ahmad Kurniawan [1], Iim Halimah [1], Maula E. Sriyani [1], Teguh H. A. Wibawa [1], Muhamad B. Febrian [1], Yanuar Setiadi [1], Eva M. Widyasari [1], Isti Daruwati [1], Crhisterra E. Kusumaningrum [1], Toto Subroto [4,5,*]

1. Research and Technology Center for Applied Nuclear, National Research and Innovation Agency, Jl. Tamansari No. 71, Lb. Siliwangi, Coblong, Bandung 40132, Indonesia; hendriswongso@batan.go.id
2. Veterinary Pharmaceutical Industry, PT. Tekad Mandiri Citra, Jl. Mekar Raya Kav. 9, Bandung 40614, Indonesia; wyandaa@gmail.com
3. Faculty of Pharmacy, Universitas Bhakti Kencana, Jl. Soekarno-Hatta No. 754, Bandung 40614, Indonesia; idar@bku.ac.id
4. Department of Chemistry, Faculty of Mathematics and Natural Sciences, Universitas Padjadjaran, Jl Raya Bandung Sumedang Km 21, Jatinangor, Sumedang 45363, Indonesia; m.yusuf@unpad.ac.id
5. Research Center for Molecular Biotechnology and Bioinformatics, Universitas Padjadjaran, Jalan Singaperbangsa No. 2, Bandung 40132
6. Department of Nuclear Medicine and Molecular Theranostics, Faculty of Medicine, Universitas Padjadjaran/Hasan Sadikin General Hospital, Jl. Pasteur No. 38, Bandung 40161; a.achmad@unpad.ac.id
7. Department of Pharmaceutical Analysis and Medicinal Chemistry, Faculty of Pharmacy, Universitas Padjadjaran, Jl. Bandung-Sumedang Km 21, Jatinangor, Sumedang 45363; holis@unpad.ac.id

* Correspondence: t.subroto@unpad.ac.id

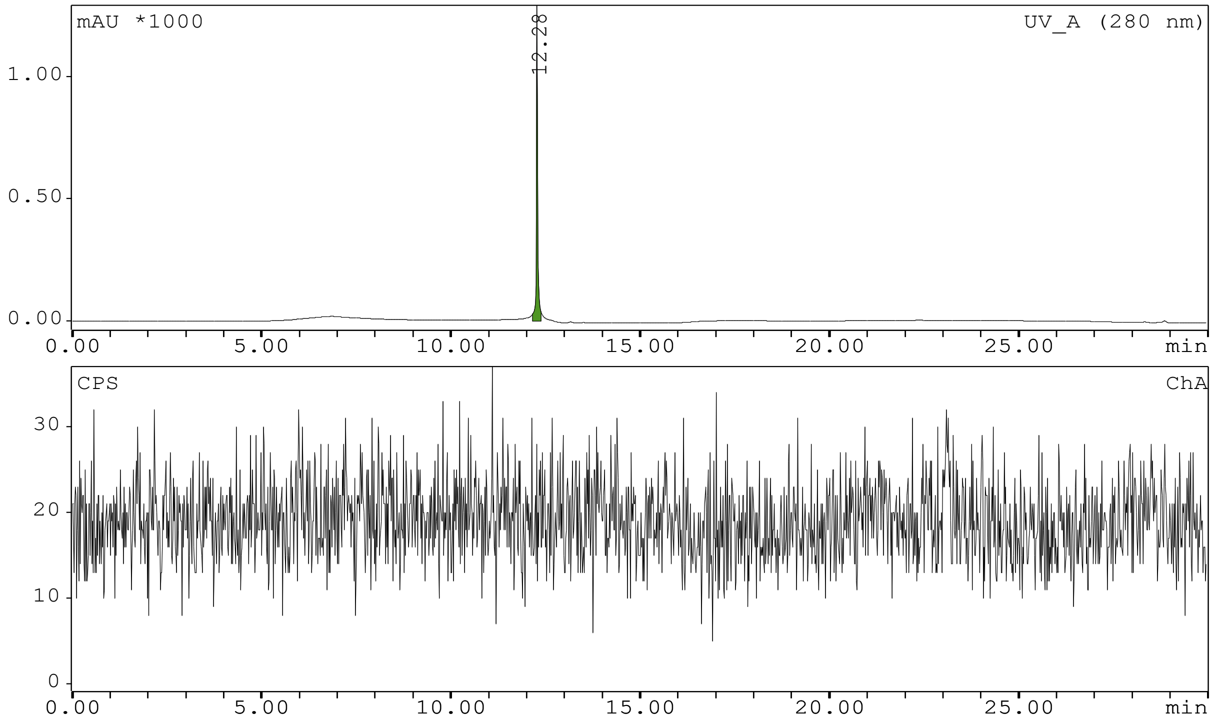

Figure S1. HPLC chromatogram of the standard IgY (RT = 12.28 min).

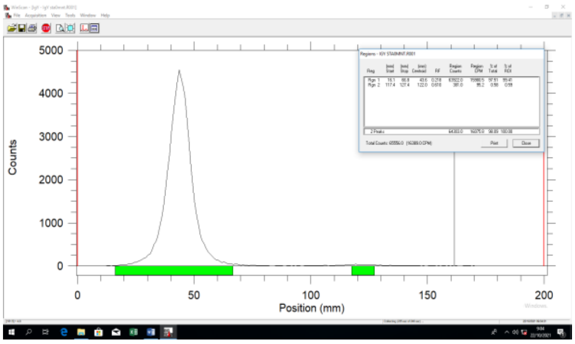

0 min

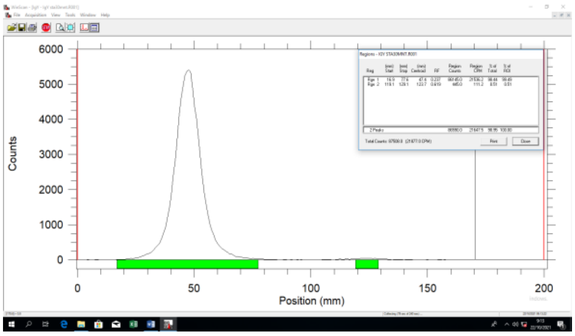

30 min

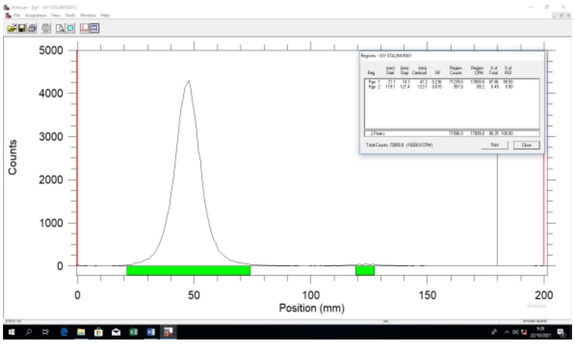

120 min

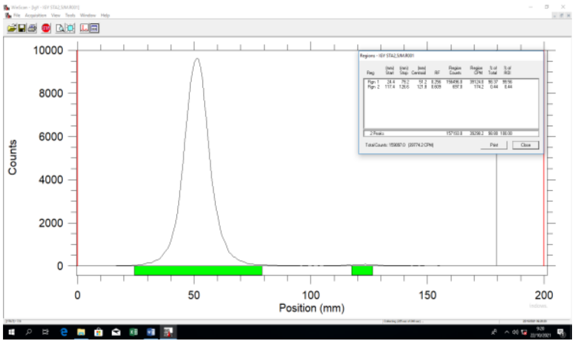

150 min

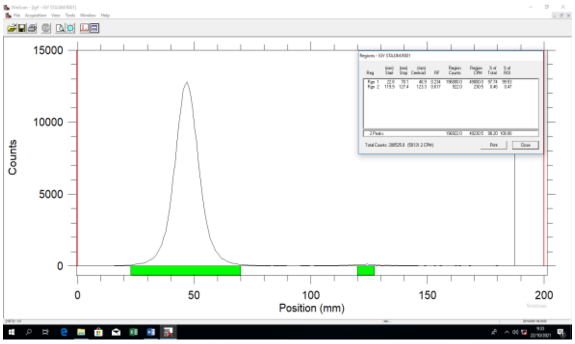

180 min

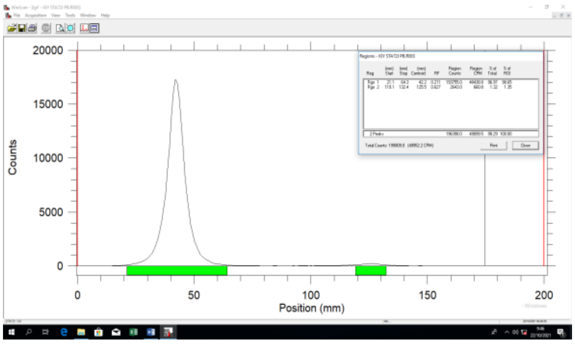

24 h

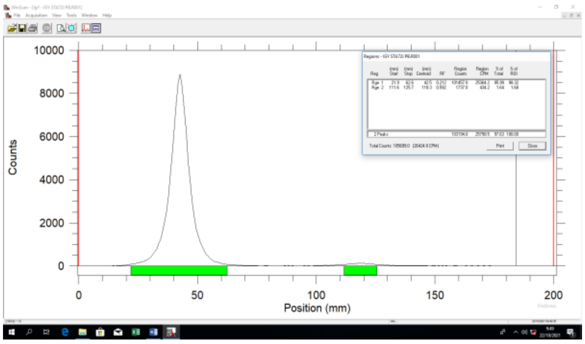

48 h

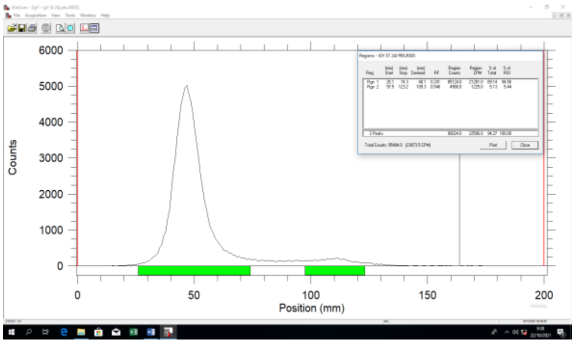

72 h

Figure S2. Representative TLC chromatograms for stability studies of [^{131}I]-IgY anti-RBD spike SARS-CoV-2 in phosphate buffer saline (PBS) over 72 h.

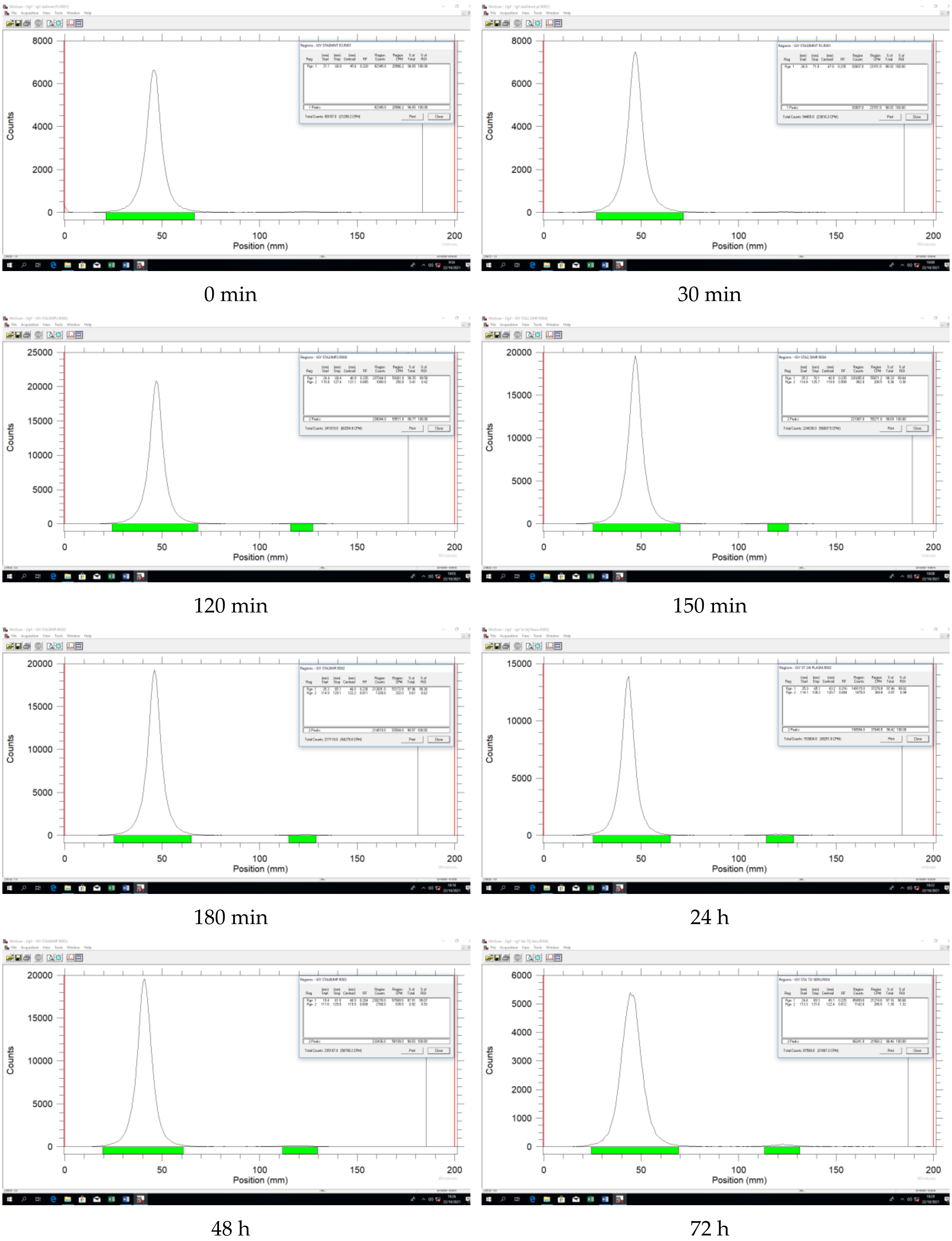

Figure S3. Representative TLC chromatograms for stability studies of [^{131}I]-IgY anti-RBD spike SARS-CoV-2 in human serum over 72 h.

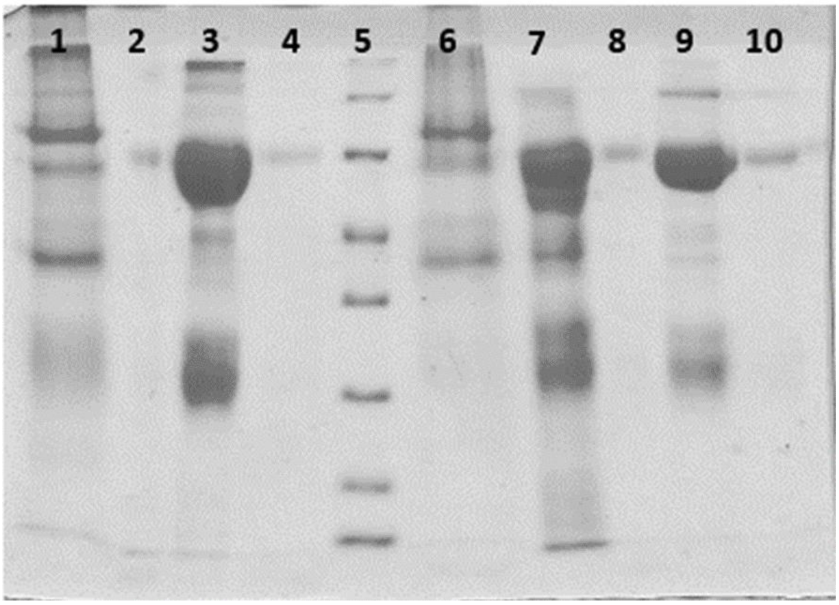

Figure S4. Identification and SDS-PAGE patterns of IgY anti-RBD spike SARS-CoV-2. Peak 1, gradient elution method (lane 1); Peak 3, gradient elution method (lane 2); ultrafiltrated peak 2, gradient elution method (lane 3); filtrate of ultrafiltrated peak 2, gradient elution method (lane 4); Protein marker (lane 5); peak 1, isocratic elution method (lane 6); peak 2, isocratic elution method (lane 7); peak 3, isocratic elution method (lane 8); ultrafiltrated of peak 2, isocratic elution method (lane 9); filtrate of peak 2, isocratic elution method.

Table S1. Lipophilicity (Log *P*) of [^{131}I]-IgY anti-RBD spike SARS-CoV-2.

**Radioactivity (count per min)**		***P***	**Log *P***
**Octanol**	**NaCl (water)**		
706	66393	0.0047	-2.33
510	72839	0.0015	-2.82
472	49271	0.0015	-2.82
		Log *P* (mean) = -2.66 ± 0.28	
